# In vivo antral follicle wall biopsy: a new research technique to study ovarian function at the cellular and molecular levels

**DOI:** 10.1186/s12958-018-0380-8

**Published:** 2018-07-28

**Authors:** G. M. Ishak, S. T. Bashir, G. A. Dutra, G. D. A. Gastal, M. O. Gastal, C. A. Cavinder, J. M. Feugang, E. L. Gastal

**Affiliations:** 1 Department of Animal Science, Food and Nutrition, Southern Illinois University, 1205 Lincoln Drive, MC 4417, Carbondale, IL 62901 USA; 2 Department of Surgery and Obstetrics, College of Veterinary Medicine, University of Baghdad, Baghdad, Iraq; 3 Department of Animal and Dairy Sciences, Mississippi State University, Mississippi State, MS USA

**Keywords:** Follicle wall biopsy, Follicular fluid, Folliculogenesis, Ovary, Horses

## Abstract

**Background:**

In vivo studies involving molecular markers of the follicle wall associated with follicular fluid (FF) milieu are crucial for a better understanding of follicle dynamics. The inability to obtain in vivo samples of antral follicle wall (granulosa and theca cells) without jeopardizing ovarian function has restricted advancement in knowledge of folliculogenesis in several species. The purpose of this study in mares was to develop and validate a novel, minimally invasive in vivo technique for simultaneous collection of follicle wall biopsy (FWB) and FF samples, and repeated collection from the same individual, during different stages of antral follicle development. We hypothesized that the in vivo FWB technique provides samples that maintain the normal histological tissue structure of the follicle wall layers, offers sufficient material for various cellular and molecular techniques, and allows simultaneous retrieval of FF.

**Methods:**

In Experiment 1 (ex vivo), each follicle was sampled using two techniques: biopsy forceps and scalpel blade (control). In Experiment 2 (in vivo), FWB and FF samples from 10-, 20-, and 30-mm follicles were repeatedly and simultaneously obtained through transvaginal ultrasound-guided technique.

**Results:**

In Experiment 1, the thickness of granulosa, theca interna, and theca externa layers was not influenced (*P* > 0.05) by the harvesting techniques. In Experiment 2, the overall recovery rates of FWB and FF samples were 97 and 100%, respectively. However, the success rate of obtaining samples with all layers of the follicle wall and clear FF varied according to follicle size. The expression of luteinizing hormone receptor (LHR) was mostly confined in the theca interna layer, with the estradiol-related receptor alpha (ERRα) in the granulosa and theca interna layers. The 30-mm follicle group had greater (*P* < 0.05) LHR expression in the theca interna and ERRα in the granulosa layer compared to the other groups. The overall expression of LHR and ERRα, and the intrafollicular estradiol were higher (*P* < 0.05 – *P* < 0.0001) in the 30-mm follicle group.

**Conclusion:**

The in vivo technique developed in this study can be repeatedly and simultaneously used to provide sufficient FWB and FF samples for various cellular and molecular studies without jeopardizing the ovarian function, and has the potential to be translated to other species, including humans.

**Electronic supplementary material:**

The online version of this article (10.1186/s12958-018-0380-8) contains supplementary material, which is available to authorized users.

## Background

Ultrasound-guided techniques for follicular fluid sampling, intrafollicular injection, retrieval of granulosa cells and oocytes, and ovarian tissue biopsy have significantly advanced knowledge of folliculogenesis in several species [1–8]. However, there is still a lack of information regarding the relationship among in vivo protein expression in the ovarian follicular wall (i.e., granulosa and theca layers), follicle growth and hemodynamics, and follicular fluid components (e.g., hormones, growth factors, proteins, and metabolites). This knowledge is not only vital for a better understanding of the mechanisms involved in follicle growth, selection, and ovulation, but can also be used to improve assisted reproductive technologies. Furthermore, studying the follicle environment during the final phase of follicular development in vivo can help in understanding mechanisms related to oocyte maturation and ovulation, which in turn may contribute to improving the in vitro oocyte maturation success rate.

The recent progress in molecular biology techniques has brought a new perspective to our knowledge in the area of mammalian folliculogenesis. Previous studies in livestock animals used antral follicles that were obtained from abattoirs, ovariectomy, or euthanasia [9–19]. Although those ovarian sources provide sufficient materials for various molecular biology techniques, follow-up studies are either impossible or potentially jeopardize animals’ fertility. In this regard, ovaries obtained from slaughterhouses have been used for developing in vitro culture systems for both granulosa and theca cells, and follicle wall culture; those systems have proven to be vital for the understanding of the follicle wall’s cell function, steroidogenesis, and, subsequently, mammalian folliculogenesis [20–22]. However, in most cases when ovaries obtained from post-mortem animals are used, the experimental design is limited and compromised by lack of control of follicle health status.

Because of the difficulty of collecting in vivo ovarian follicular wall samples from living animals without jeopardizing the animals’ fertility or the study design, there is a necessity to develop a minimally invasive technique to harvest follicular wall biopsy (FWB) and follicular fluid (FF) samples for research and diagnostic purposes. This technique could be helpful for repeated in vivo sampling of the follicle wall and FF of the same individual simultaneously, without disturbing ovarian function. Furthermore, this technique would provide a better understanding of physiological and pathological mechanisms involved in ovarian function, such as follicle selection and dominance, and formation of the hemorrhagic anovulatory follicle (HAF) or luteinized unruptured follicle (LUF) syndrome, and ovarian cyst in different species [23–27].

In the past two decades, considerable attention has been given to the importance of mares as a comparative research model to study ovarian folliculogenesis in women (for review see [28]). Several studies have shown striking similarities between mares and women in follicle dynamics, ovulatory follicular wave, ovulation dysfunction, and reproductive aging process [23, 29–33]. Additionally, the advantage of using the equine follicle is that the diameter of follicles in mares is more than twice the diameter of follicles in women [33], providing, therefore, an excellent in vivo source for developing and validating a new technique to collect ovarian follicle wall samples. The success of harvesting from the mare in vivo sufficient ovarian follicle samples for various experimental techniques (e.g. histology, cell, and molecular biology) will give the opportunity to adapt the FWB procedure to other species, including humans. Hence, the use of mares in such studies will provide a dual benefit to advance knowledge in reproductive biology of farm animals and potentially in women as well.

The aims of this study in mares were to: (i) develop ex vivo a minimally invasive technique for harvesting of FWB samples (Experiment 1); (ii) validate the FWB technique for simultaneous in vivo collection of FWB and FF samples (Experiment 2); and (iii) characterize the expression of luteinizing hormone receptor (LHR) and estradiol-related receptor alpha (ERRα) in in vivo harvested follicle wall layers, and intrafollicular estradiol concentration during different follicle developmental stages (Experiment 2). The hypotheses tested were: (i) in vivo FWB allows obtaining sufficient material for various experimental techniques; (ii) samples of FWB obtained in vivo maintain normal histological tissue structure of the follicular wall layers independent of follicle size; and (iii) in vivo FWB technique allows simultaneous collection of clear FF samples.

## Methods

The current study was carried out in two experiments: ex vivo follicle wall biopsy (Experiment 1; performed at Mississippi State University, Mississippi State, USA), and in vivo follicle wall biopsy (Experiment 2; performed at Southern Illinois University, Carbondale, IL, USA).

### Experiment 1. Ex vivo follicle wall biopsy

#### Animals and ovarian follicles

Ovaries of six mares, 6–18 years old, were collected from animals euthanized at Mississippi State University during the transitional anovulatory season (February) to develop the FWB technique. For the euthanasia procedure, mares were sedated and anesthetized using xylazine (1.1 mg/kg BW) and ketamine (2.2 mg/kg BW), respectively; thereafter, potassium chloride (KCl) solution was injected to cease cardiac function. Ultrasonographic examinations were carried out on the day before euthanasia to determine the follicle population in each animal. Immediately after euthanasia, ovaries were harvested and transported to the laboratory in an icebox. Antral follicles were located and measured macroscopically using a traceable digital caliper (Fisher Scientific, St. Louis, MO, USA), and matched with the ultrasound follicle mapping information carried out on the day before euthanasia. Thereafter, follicles were divided into small (8–10 mm), medium (11–19 mm), and large (20–29 mm) groups.

#### Follicle wall biopsy and scalpel blade sampling

**Fig. 1:**
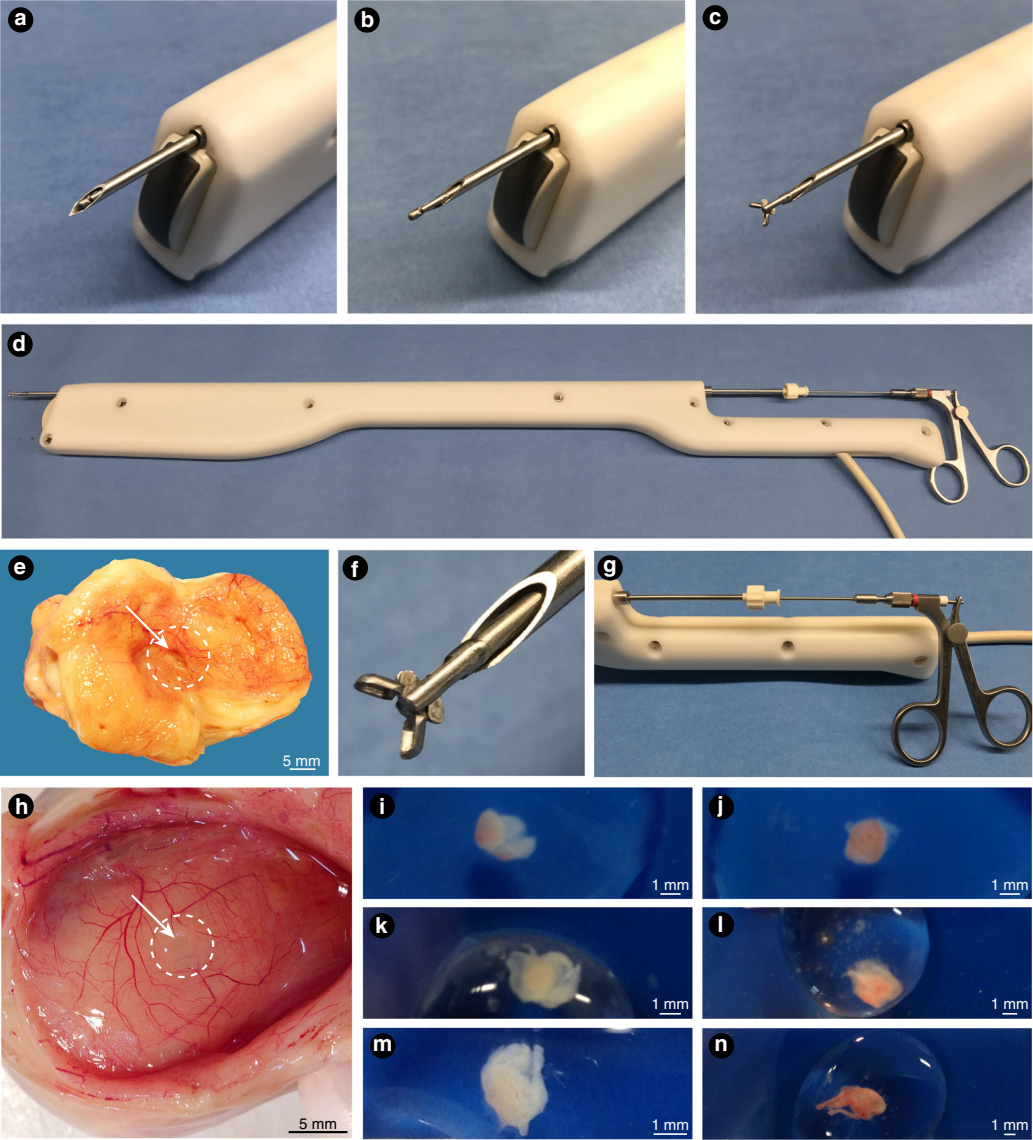
Transvaginal ultrasound transducer used for the follicle wall biopsy (FWB) procedures. **a–d** Biopsy forceps located inside of a 12 G needle/ cannula and advancing through the needle guide of the transvaginal ultrasound probe handle; **e** biopsy mark (arrow) in the wall of a 29-mm follicle after harvesting the biopsy sample; **f** close-up of the tip of the biopsy forceps opened within the cannula; **g** handle of the transvaginal ultrasound probe showing the biopsy forceps within the cannula and introduced through the needle guide; **h** biopsy mark (arrow) in the wall of a 27-mm follicle after harvesting the biopsy sample; **e** and **h** the vascularity of the follicle wall is evident in both follicles; **i − n** typical FWB samples obtained in vivo from different follicle sizes

Biopsy of the follicular wall was carried out using an endoscopic biopsy forceps (5 FR gauge, 60 cm, MPN# 27425Z, Karl Storz, Berlin, Germany; Fig. 1) located inside of a 12 G needle/ cannula (length 53 cm; MOFA Global, Verona, WI, USA). After the needle perforated the follicle wall, it was retracted back to the extent that only the tip and bevel of the needle were kept inside. Then, the biopsy forceps was propelled forward and the tip was opened inside the follicle. The open tip of the biopsy forceps was further pushed to make firm contact with the side of the follicle wall, opposite to the side of entry of the needle. Thereafter, the tip of the biopsy forceps was closed and pulled back to harvest the FWB sample. Afterward, the biopsy forceps with the needle was retracted out of the follicle. Immediately after the biopsy procedure, the tip of the biopsy forceps was opened, and the biopsy sample was carefully removed from the forceps and placed into a petri dish containing phosphate buffered saline (PBS). From each follicle, two FWB samples were obtained using the method. As soon as the FWB samples were taken, the follicle was opened in two halves and a third sample of follicle wall was obtained using a scalpel blade (control group). The FWB and follicle scalpel samples were placed in a petri dish with PBS solution and a picture was taken for estimating the area and perimeter of the samples using the ImageJ software (version 1.50f). Samples’ weights were recorded using an analytical balance (TR-204, Denver Instruments, Denver, CO, USA). Then, samples were immediately processed for standard histological analyses.

### Experiment 2. In vivo follicle wall biopsy

#### Animals and ovarian follicles

The study was carried out in the northern hemisphere (latitude, 37.7°N; longitude, 89.2°W), using 18 Quarter Horse mares, 8–14 years old, and weighing 400–550 kg during the spring season (March to May).

#### Ultrasonography, follicle tracking, and groups

All ovarian follicles ≥6 mm were ablated by ultrasound-guided transvaginal aspiration of follicular contents to induce a new follicular wave as previously described [34]. Then, ultrasonographic follicle tracking was performed daily using a duplex color-Doppler ultrasound machine (Aloka SSD-3500; Hitachi Aloka Medical America, Inc., Wallingford, CT, USA) equipped with a finger-mounted 3.5–10 MHz convex-array transducer (UST-995-7.5). Follicles observed growing for at least three consecutive days were classified into: small (10–14 mm), medium (20–24 mm), and large (30–34 mm) follicle groups.

#### In vivo follicle wall biopsy procedure

Mares were prepared and sedated as previously described [5]. Briefly, for each FWB procedure, mares were placed in a palpation chute to eliminate excessive movement; the mare’s rectum was evacuated and the tail was wrapped in a palpation sleeve to decrease the chance of contamination. The perineal area was aseptically prepared through washing with povidone-iodine solution and alcohol; thereafter, the mare’s urinary bladder was cannulated with a Foley catheter. Ten minutes before the start of each FWB procedure, rectal relaxation and analgesia were induced with hyoscine N-butyl bromide (Buscopan, 0.2 mg/kg i.v.; Sigma, St. Louis, MO, USA) and flunixin meglumine (Flunixiject, 1.1 mg/kg i.v.; Butler Schein Animal Health, Dublin, OH, USA), respectively. Five minutes before the start of the FWB procedure, sedation and analgesia were induced with xylazine (AnaSed, 1 mg/kg i.v.; Lloyd Laboratories, Shenandoah, IA, USA) and detomidine (Dormosedan, 0.02–0.04 mg/kg i.v.; Zoetis, Florham Park, NJ, USA), respectively.

**Fig. 2:**
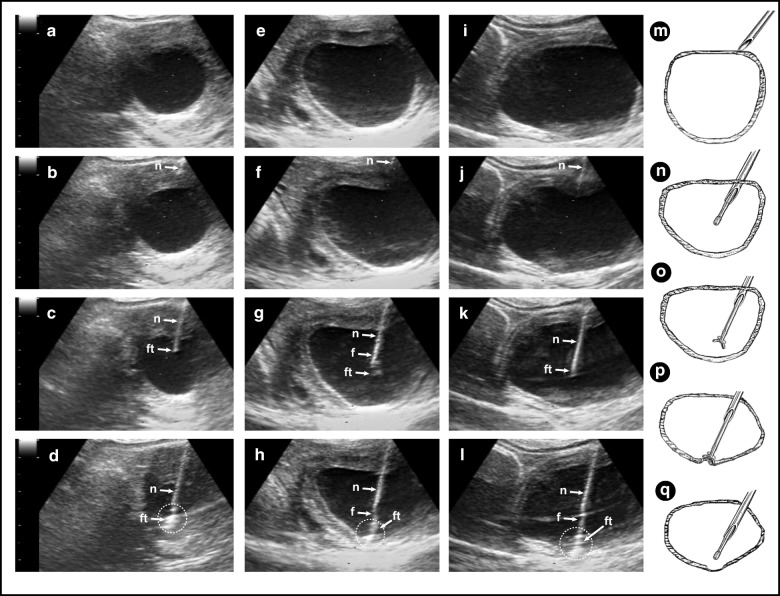
Transvaginal ultrasound images during three follicle wall biopsy (FWB) procedures. **a–d** ovary with a small follicle size; **e–h** ovary with a medium follicle size; **i–l** ovary with a large follicle size; **a, e,** and **i** follicles ready to be biopsied; **b, f,** and **j** the 12 G needle/ cannula can be seen within the ovary but outside the follicle; **c, g,** and **k** the cannula and the biopsy forceps advance into the follicle antrum; **d, h,** and **l** biopsy forceps in close contact with the follicle wall to harvest the FWB tissue; **m-q** diagram illustrates steps of the FWB procedure; **m** cannula outside the ovary; **n** cannula inside the follicle antrum showing the extended tip of the biopsy forceps closed; **o** biopsy forceps opened inside the follicle; **p** biopsy forceps harvesting the FWB sample and bulging inside the follicle wall; **q** biopsy forceps being retrieved closed toward the cannula; a mark in the follicle wall can be seen sometimes immediately after pulling back the biopsy forceps. Simultaneously after the retrieval of the biopsy forceps from the cannula, a clean sample of follicular fluid is obtained immediately. n, needle, f, forceps; ft., forceps tip


Follicle wall samples were obtained using an endoscopic biopsy forceps, as described in Experiment 1, with the exception that only one FWB was obtained from each follicle. The biopsy forceps covered by the 12 G needle was introduced into a needle guide mounted on a 5–10 MHz transvaginal ultrasound-guided convex array transducer (Aloka UST-987-7.5; Fig. 1a-d, f-g). Once the targeted follicle was displayed on the ultrasound screen, the needle was introduced into the antrum of the follicle and a second operator harvested the FWB sample (Fig. 2). At this point, only the biopsy forceps was retracted back, while the tip of the needle was kept inside the follicle to collect a FF sample. A diagram demonstrating the FWB procedure is provided (Fig. 2m-q). In several instances, mares with more than one targeted growing follicle, which fitted within the small and medium follicle groups, were biopsied during the same procedure. After FWB samples were obtained from the three follicle classes in each mare, daily ultrasonography examination was performed to monitor the effect of the FWB sampling technique on the continuation of the ovarian activity. Images of typical FWB samples obtained in vivo are shown (Fig. 1i-n). Two additional movie files show the FWB technique being performed in medium and large follicle sizes (Additional file 1: Video clip S1; Additional file 2: Video clip S2).

**Additional file 1: Video clip S1.** Transvaginal ultrasound-guided follicle wall biopsy procedure in a 30-mm follicle, with simultaneous follicular fluid harvesting. (MP4 37096 kb)



**Additional file 2: Video clip S2.** Transvaginal ultrasound-guided follicle wall biopsy procedure in a 24-mm follicle, with simultaneous follicular fluid harvesting. (MP4 47271 kb)


#### In vivo follicular fluid sampling

After in vivo harvesting the FWB sample, FF was collected by a third operator while the needle was still inside the follicle. For small and medium size follicles, a 3 ml syringe attached to a 5 cm silicone tube and luer locks was immediately connected to the 12 G needle. For large size follicles, leaking FF from the needle was collected directly into a petri dish. Immediately after collection, FF samples were centrifuged (1500 *g* for 10 min, at 4 °C) and supernatants were stored at − 80 °C until analyses.

### Histology process and evaluation

In Experiment 1, two harvested samples (1 biopsy and 1 scalpel) were collected from each follicle (*n* = 47) and fixed in Bouin’s solution for 2 h. Thereafter, fixed samples were transferred to 70% ethanol and stored at 4 °C until standard histological processing [35]. Samples were embedded in paraffin wax and cut into 5 μm serial sections using a rotary microtome (model 2030; Leica Instruments GmbH, Germany); every section was mounted and stained with Periodic Acid-Schiff (PAS) and counterstained with hematoxylin. In Experiment 2, FWB samples (one from each follicle) were fixed in 10% formalin solution and processed as described in Experiment 1. Histological sections were analyzed using a light microscope (Nikon E200, Tokyo, Japan) at 20× objective magnification and an image capture system (LEICA Imaging Software, Wetzlar, Germany).

### Measurements of the follicle wall layers

In Experiment 1, two histological sections per sample were chosen to measure the thickness of each layer of the follicle wall (granulosa, theca interna, and theca externa) as previously described [36, 37]. Briefly, granulosa and theca interna layers were identified based on the visualization of the follicle basement membrane. The granulosa layer was characterized by flattened cells extending from the basement membrane to the follicle antrum, while the theca interna layer showed endocrine-like cells extending outward from the basement membrane until the beginning of the theca externa layer. The theca interna layer was further identified by high numbers of blood vessels. A wavy appearance characterized cells of the theca externa, similar to fibroblast cells. Three measurements were randomly taken for each layer within each sample section, with the average data considered as the thickness for each layer.

### Immunohistochemistry

The presence of LHR and ERRα (Experiment 2) in follicle wall layers was assessed using immunofluorescence detection as previously described [38, 39]. Briefly, sample sections were deparaffinized, submitted to antigen retrieval (sodium citrate buffer for 30 min in a steamer), permeabilized (1% Triton X-100 for 30 min), blocked (PBS-1% BSA for 60 min), and incubated (60 min) with respective primary antibodies at 1:100 dilution (LH: LS-C312710, ERRα: LS-A5402, LifeSpan BioSciences, Seattle, WA). Thereafter, sections were immersed with the goat anti-rabbit secondary antibody FITC conjugated (dilution, 1:200) and incubated in the dark (60 min). Sections were immediately immersed with a DAPI-contained mounting medium to counterstain all sample nuclei (blue-stained). All steps were performed at room temperature, and sections were washed three times with PBS between all steps. Negative controls consisted of sample sections incubated with PBS without the primary antibodies. Mounted sections were subsequently subjected to epifluorescence microscope imaging (EVOS FL-Auto Cell Imaging System, Thermo Fisher Scientific Inc., Waltham, MA) to assess the presence of targeted proteins appearing green (FITC stained). The LHR primary antibody has been successfully used in a previous study with equine tissues [40]. Despite the manufacture’s claim of 100% epitope sequence identity with the equine ERRα protein, we performed a Western immunoblotting to further validate the specificity of the ERRα antibody with the mare’s ovary tissue before the immunohistochemistry (IHC) analysis, using a previously described procedure [38].

Image analyses focused on the overall (entire) and layer-specific (granulosa, theca interna, and theca externa cells) follicle wall expression of LHR and ERRα proteins. Each follicle wall layer was precisely delimited with the freehand tool of the ImageJ software. Both FITC (green) and DAPI (blue) fluorescence signals were recorded for each overall or layer-specific follicle wall. To allow a direct comparison among the follicle wall layers, the expression of LHR and ERRα levels was determined as the proportions of green over blue signals (× 100).

### Estradiol analysis

In Experiment 2, intrafollicular estradiol concentration was measured in each follicle using the equine ELISA kit (Endocrine Technologies, Inc., Freemont, CA, USA) as recommended by the manufacturer. Samples were diluted 1:500–1:3000 in assay buffer, and the intra-assay CV was 9.0%, while the sensitivity of the assay was 10 pg/ml. The plate was read using the SpectraMaxPlus Microplate Reader (Molecular Devices, LLC, CA USA), and the data were analyzed by the software SoftMax Pro 6.5.1.

### Statistical analyses

Shapiro-Wilk test was used to evaluate the normal distribution of the dataset; data that were not normally distributed were transformed to either rank or log. Dixon’s test was used to identify outlier observations, which were excluded from any statistical analysis. Comparisons among multiple groups were performed by one-way ANOVA, followed by Tukey’s test if a significant difference was detected. Student’s *t* test was used to compare the data between two groups. All the statistical analyses were done using JMP software version 13.0 (SAS Institute Inc., Cary, NC, USA). Data were expressed as mean ± SEM, unless otherwise indicated. A probability of *P* < 0.05 indicated that a difference was significant, and *P* > 0.05 and ≤ 0.1 indicated that a difference approached significance.

## Results

### Experiment 1. Ex vivo follicle wall biopsy

**Fig. 3:**
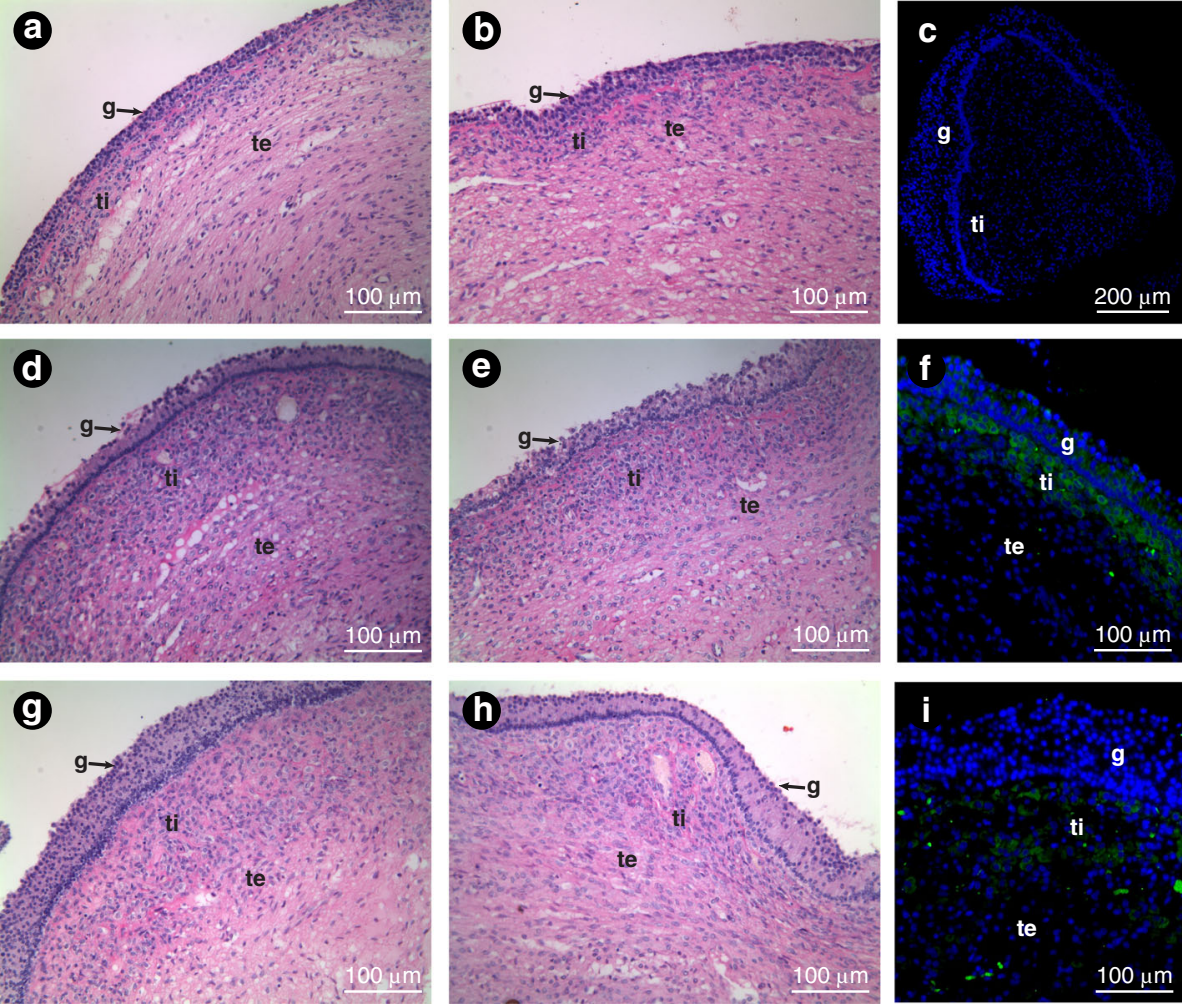
Examples of histological sections of ex vivo follicle walls (Experiment 1) obtained from (**a** and **b**) small, (**d** and **e**) medium, and (**g** and **h**) large follicles using the FWB (**a, d, g**), and scalpel blade (**b, e, h**) techniques. For comparative purposes, the same follicle was sampled twice, i.e., with the FWB technique and the scalpel method. **c, f, i** immunofluorescence detection in walls of large follicles; **c** negative control; **f** ERRα; and (**i**) LHR. Granulosa, theca interna, and theca externa layers can be seen in the follicle wall samples. g, granulosa; ti, theca interna; and te, theca externa

**Fig. 4:**
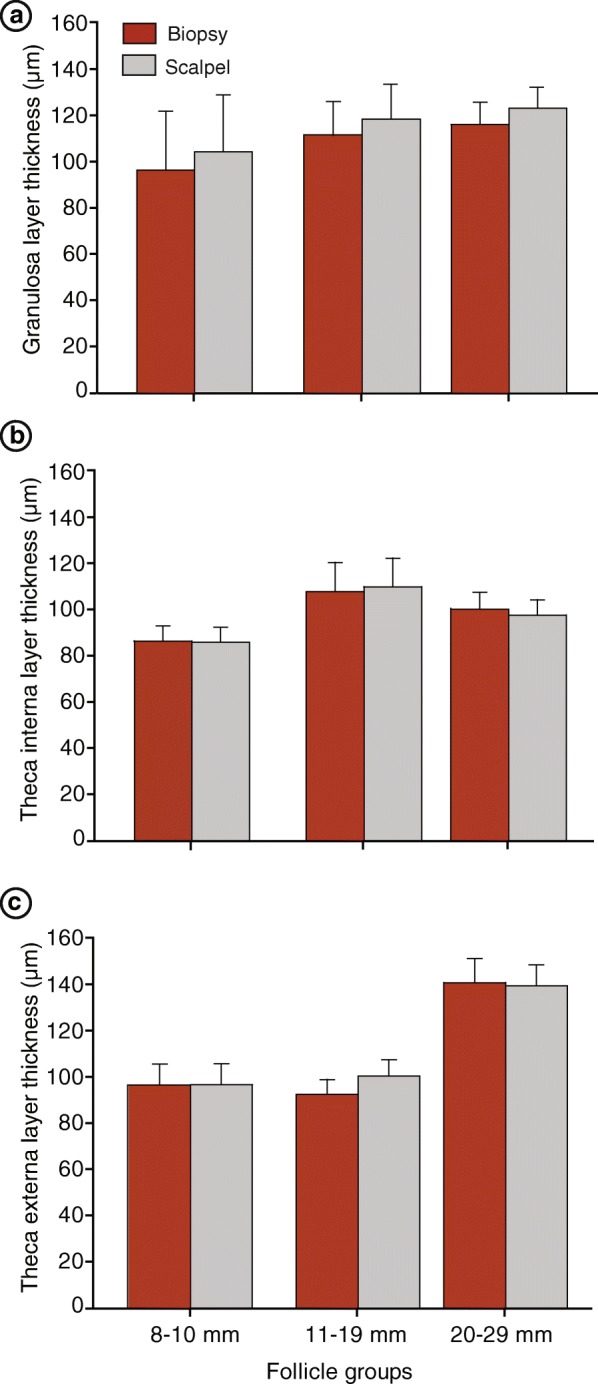
Mean (±SEM) thickness of the follicle wall layers obtained using the biopsy forceps technique versus the scalpel blade method in different follicle size groups. **a** granulosa, **b** theca interna, and (**c**) theca externa layers. No difference was observed within the same follicle size group between the approaches used to harvest the follicular wall samples

A total of 47 follicles (≥8 mm) were sampled using two techniques: biopsy forceps and scalpel blade (sample area size ~ 6–8 mm^2^). The recovery rate of the ex vivo FWB samples with only one attempt was 100%. Samples obtained using the biopsy forceps measured 6.5 ± 0.5 mm^2^ in area, 12.0 ± 0.5 mm in perimeter, and weighed 5.4 ± 0.4 mg. The histological appearance and organization of the follicle wall tissue was not affected by the FWB technique compared with the scalpel blade (Fig. 3). Furthermore, the mean thickness of each follicle wall layer (i.e., granulosa, theca interna, and theca externa) was not influenced (*P* > 0.05) by the technique used to harvest the samples (Fig. 4). The FWB technique has also proved to be efficient to obtain follicle wall samples from follicles as small as 8 mm without affecting the quality of the tissue sample obtained.

### Experiment 2. In vivo follicle wall biopsy

**Fig. 5:**
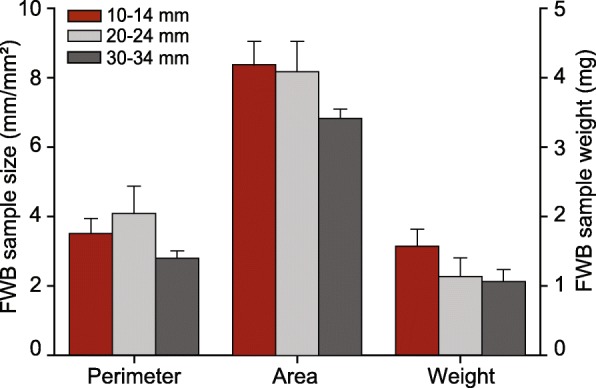
Mean (±SEM) perimeter, area, and weight of follicular wall samples harvested using the in vivo follicle wall biopsy technique in different follicle size groups. No difference was observed among follicle groups within each endpoint

Follicle wall and FF samples were collected simultaneously from the same mares using our novel biopsy sampling technique. A total of 72 follicle wall and FF samples were obtained from the three follicle groups, as follows: 10–14 mm (*n* = 34), 20–24 mm (*n* = 20), and 30–34 mm (*n* = 18). Overall, the recovery rate of FWB samples was 97.3%. The in vivo FWB samples measured 3.5 ± 0.3 mm^2^ in area, 7.9 ± 0.4 mm in perimeter, and weighed 2.6 ± 0.3 mg. The diameter of the follicle did not influence (*P* > 0.05) the area, perimeter, or weight of the FWB samples harvested (Fig. 5). The presence of the granulosa, theca interna, and theca externa layers in all FWB samples was 72.2, 88.8, and 91.6%, respectively. Furthermore, the percentage of samples with the three follicle wall layers in 10-, 20-, and 30-mm follicles was 58.8, 80.0, and 78.8%, respectively. FWB samples with the presence of three, two, or only one follicle wall layer were 69.5, 18.0, and 12.5%, respectively. The percentage of samples without the granulosa cell layer tended (*P* = 0.07) to be greater in the 10-mm than in the 20-mm follicles, but did not differ from 30-mm follicles. The samples with clear FF were 83.3%; however, it was greater (*P* < 0.05) in 30-mm follicles (100%) compared with 10-mm follicles (70.6%), with no difference between 20-mm (90%) and 10-mm follicles. In vivo antral follicle wall sampling using the FWB technique did not reveal any detrimental effect on the ovarian function; all mares ovulated on average 24.7 ± 3.3 days after the last biopsy procedure. Furthermore, mares were allocated to other studies and continued to cycle regularly until the end of the reproductive season.

#### Immunohistochemistry

**Fig. 6:**
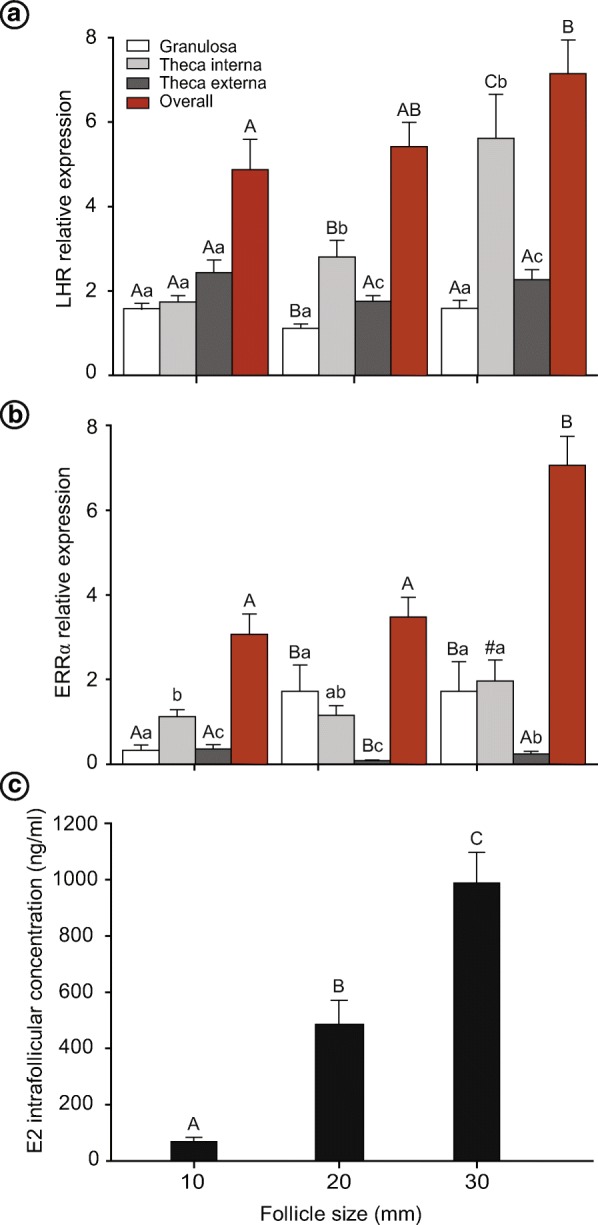
Mean (±SEM) (**a** and **b**) immunofluorescence relative expression of (**a**) LHR, and (**b**) ERRα in the granulosa, theca interna, theca externa, and in all layers of the follicle wall (overall); and (**c**) intrafollicular estradiol concentration in 10-, 20-, and 30-mm follicle groups. E2, estradiol. ^a,b,c^ Within follicle size groups, follicle layers without a common superscript differed (*P* < 0.05). ^A,B,C^ Among follicle size groups, follicle layer without a common superscript differed (*P* < 0.05). ^#^ Tended (*P* = 0.06) to differ from the 10-mm follicle group

A higher (*P* < 0.05) expression of LHR in the granulosa cells of 30- and 10-mm follicles was detected compared with the 20-mm follicles (Fig. 6a). Expression of LHR in the theca interna layer increased (*P* < 0.05), along with an increase in follicle diameter. The lowest expression of LHR was seen in the theca interna of 10-mm follicles, followed by 20-mm follicles, with the highest expression seen in the 30-mm follicles. However, no difference (*P* > 0.05) was observed in LHR expression in the theca externa of different follicle groups. The overall expression of LHR in all follicle wall layers was higher (*P* < 0.05) in the 30-mm follicles compared with the 10-mm follicles. Additionally, the expression of LHR was confined mostly (*P* < 0.05 - *P* < 0.01) to the theca interna layer of the 20- and 30-mm follicles compared with the granulosa and theca externa layers of the same follicle groups.

The expression level of the ERRα was higher (*P* < 0.05) in the granulosa cells of 20- and 30-mm follicles compared with 10-mm follicles (Fig. 6b). Furthermore, the ERRα expression in the theca interna of the 30-mm follicles tended (*P* = 0.06) to be higher compared with the 10-mm follicles. The overall expression of ERRα in all follicle wall layers was higher (*P* < 0.05) in the 30-mm follicles compared with the 20- and 10-mm follicles. Regardless of follicle size, the expression of ERRα was stronger (*P* < 0.05 - *P* < 0.01) and mostly confined to the granulosa and theca interna layers of 20- and 30-mm follicles compared with the theca externa of the same follicle groups.

#### Intrafollicular estradiol

Follicular fluid concentration of estradiol increased (*P* < 0.001), along with an increase in follicle diameter (Fig. 6c). Intrafollicular estradiol concentration was greater (*P* < 0.0001) in 30-mm follicles compared with the 20- and 10-mm follicles. In addition, estradiol concentration was greater (*P* < 0.001) in the 20-mm compared with the 10-mm follicles.

## Discussion

This is the first report on the development of an in vivo technique to collect antral FWB samples in any species. Two experiments were conducted to develop and validate the FWB technique to collect samples from living mares. Samples of follicle walls from antral follicles have been harvested previously using materials obtained from slaughterhouses [14, 41], animals sacrificed [13, 19, 42, 43], or through invasive surgeries such as ovariectomy [9, 15, 16, 18, 44, 45]. However, in most cases the inability to determine the follicle health status and the impossibility of follow-up studies are major factors that limit more in-depth advancement in knowledge regarding follicle dynamics and ovulation in different species. Therefore, there is a necessity for alternative minimally invasive techniques to harvest FWB samples. In addition to the ability to harvest FWB samples repeatedly from the same individual during different phases of the follicular wave, using ultrasound tracked follicles, the FWB technique developed in this study allows simultaneous collection of FF samples from the same biopsied follicle. Hence, the implementation of the FWB technique will provide in vivo materials for various cellular and molecular biology studies associated with FF milieu, follicle growth, and hemodynamics. Furthermore, results of repeated FWB sampling using the same mares showed no detrimental effect on ovarian function or estrous cyclicity. A follow-up ultrasound examination revealed normal resumption of follicle development and ovulation soon after the last FWB procedure. In addition, repeated FWB has the potential to be used to compare ovarian pathological conditions such as HAF/ LUF versus normal follicle development within the same individual. The success of developing the FWB technique in mares might also be applicable to other species, including humans.

Scalpel blade sampling method has been used previously to collect ex vivo follicle wall samples [9, 13, 14, 16, 18, 19, 45]. Therefore, in Experiment 1, the scalpel blade was used as a control group of the FWB technique to compare the tissue quality of the same follicle obtained by both techniques. Although the FWB samples were small in size, because of the small jaws of the biopsy forceps, the histological morphology and thickness of the FWB layers (granulosa, theca interna, and theca externa) were similar to the scalpel blade regardless of the follicle size. Furthermore, these results clearly demonstrated that the pulling force used to harvest the FWB samples had no harmful effect on the biopsy samples; therefore, the samples obtained through the FWB technique were equally representative of the follicle wall when compared with the scalpel blade. One of the limitations of the FWB technique, as a result of the diameter of the 12 G needle used to puncture the follicle, was that only follicles ≥8 mm were able to be biopsied.

To validate the FWB technique in vivo (Experiment 2), the mare was used as an animal model because of the larger preovulatory follicle diameter (≥30 mm) compared to cows (16 mm) and women (20 mm) [28, 33]. Therefore, equine follicles provide an excellent source of materials to validate the in vivo FWB technique, which may be applied later to other species. Furthermore, the larger body size and easy access to the ovaries make the mare an important animal to test transvaginal ultrasound-guided procedures with minimal stress [1–8, 46]. Additionally, mares and women have striking similarities in follicle dynamics, ovulation dysfunction, and reproductive aging process [23, 30, 32, 33, 47, 48]; these reasons reinforced the importance of the use of mares as a model in the present study.

In Experiment 2, after the induction of a new follicular wave by ablating all follicles ≥6 mm in diameter, samples were collected only from follicles that had been growing for at least three consecutive days. Although we were able to obtain follicle wall tissue in 97.3% of the attempts, the success rate of obtaining in vivo FWB samples with all follicle wall layers differed between follicle sizes; the small (10–14 mm) follicles had lower recovery rate than the larger follicles (30–35 mm). In this regard, small follicles have a smaller antrum and less volume of FF compared to large follicles, and therefore, limited space is available inside small follicles to open the biopsy forceps to harvest the FWB sample. Likewise, in small follicles a shorter time is available to biopsy the follicle before leakage of the whole FF and disappearance of the antrum occurs on the ultrasound screen. Regarding follicle wall layers, the granulosa layer was the most detached layer compared to the theca interna and externa layers. These results can be attributed to intraovarian factors; for instance, the theca interna layer is composed of connective tissue and the theca externa of fibrous tissue, with both theca layers providing structural support to the follicle [49, 50]. Therefore, these two layers are characterized by firmer tissue structure compared to the granulosa layer. In addition, the success of obtaining all follicle wall layers might also be influenced by extraovarian factors, such as animal movement during the procedure, and the experience of the operator performing the FWB procedure. Therefore, operator training and efficient animal sedation during the procedure seem to be essential to guarantee a satisfactory success rate of obtaining all layers of the follicle wall.

In the present study, to validate the in vivo FWB technique, IHC expression of LHR and ERRα was studied in all layers of the follicle wall, and estradiol concentration in FF samples obtained from different follicle sizes. The expression of the LH receptors was mainly confined to the cells of the theca interna compared to the cells of the granulosa and theca externa layers. In this regard, a previous study [18] reported that LHr mRNA was expressed in both the cells of granulosa and theca interna of preovulatory follicles, with greater expression in cells of the theca interna compared to the granulosa cells. In the present study, the 30-mm follicles (preovulatory follicles) showed a higher expression of LHR in the granulosa and theca interna, and in the overall quantification. Results of previous reports [51, 52] have shown lower expression of LH receptors on granulosa, theca, and cumulus cells of small follicles compared to larger ones; therefore, the increase in LH receptors has been correlated with follicle growth, oocyte competence, and ovulation in mares. Also, previous reports [53, 54] have shown that systemic LH is essential after deviation for establishment of dominance and further development of the largest follicle. Taken together, the results of these studies with our current finding provide considerable evidence regarding the relationship between LHR up regulation in the follicle wall and systemic LH levels during follicle development. Therefore, a comprehensive study is warranted to elucidate the expression pattern of LHR in the follicle wall with systemic and intrafollicular concentration of LH within the same individuals in relation to follicle selection and development of the dominant follicle in different species.

Results of ERRα quantification showed a greater expression in granulosa cells and in the overall quantification of 20- and 30-mm follicles compared with the 10-mm follicles; however, the ERRα expression in the cells of theca interna tended to be higher in the 30-mm compared with the 10-mm follicles. These novel results showed a clear relationship between the increase in follicle wall ERRα expression and continued follicle growth. Intrafollicular estradiol concentration increased in the present study with the increase in follicle diameter. Similar results have been reported previously in mares [55, 56]. Estrogen has been shown to induce the expression of ERRα [57], and, therefore, clearly aligns with our observation of increased estradiol as follicle diameter enlarged. However, more in-depth in vivo studies are required to elucidate the expression pattern of ERRα in relation to follicle selection and development in mares. Also, ERRα expression increases in ovarian cancers [58], which may support the use of the mare as a relevant model for studying ovarian pathophysiology. Therefore, the results of the IHC and FF analyses revealed that the materials collected with the FWB technique had similar quality to the materials obtained in previous studies using different methods, with the major advantage of using in vivo biopsy of ultrasound-tracked follicles with known physiological status. In addition, samples harvested by the in vivo FWB technique provided sufficient materials for histological and molecular biology techniques (e.g., IHC and Western blotting), and also clear FF samples that can be used for various studies including hormonal analyses and omics. Furthermore, another importance of the FWB technique is the ability to re-biopsy the same mares to compare folliculogenesis during different seasons of the year, study different pathological conditions such as HAF/ LUF syndrome, or test different treatment protocols without sacrificing the animals.

## Conclusions

In conclusion, the FWB technique developed for mares can be used repeatedly and simultaneously to harvest in vivo FWB and FF samples with a satisfactory recovery rate without jeopardizing ovarian function. This technique provides sufficient materials for various cellular and molecular studies associated with FF milieu, and has the potential to be translated to other species, including humans. The successful application of this technique may bring in a new era for in vivo folliculogenesis studies.

## Abbreviations

ERRα: Estradiol-related receptor alpha
FF: Follicular fluid
FWB: Follicle wall biopsy
HAF: Hemorrhagic anovulatory follicle
IHC: Immunohistochemistry
LHR: Luteinizing hormone receptor
LUF: Luteinized unruptured follicle
PAS: Periodic acid-Schiff
PBS: phosphate buffered saline
